# Simulating two-sided mobility platforms with MaaSSim

**DOI:** 10.1371/journal.pone.0269682

**Published:** 2022-06-09

**Authors:** Rafał Kucharski, Oded Cats

**Affiliations:** 1 Department of Transport & Planning, TU Delft, Delft, The Netherlands; 2 Faculty of Mathematics and Computer Science, Jagiellonian University, Kraków, Poland; The University of Alabama, UNITED STATES

## Abstract

Two-sided mobility platforms, such as Uber and Lyft, widely emerged in the urban mobility landscape. Distributed supply of individual drivers, matched with travellers via intermediate platform yields a new class of phenomena not present in urban mobility before. Such disruptive changes to transportation systems call for a simulation framework where researchers from various and across disciplines may introduce models aimed at representing the complex dynamics of platform-driven urban mobility. In this work, we present MaaSSim, a lightweight agent-based simulator reproducing the transport system used by two kinds of agents: (i) travellers, requesting to travel from their origin to destination at a given time, and (ii) drivers supplying their travel needs by offering them rides. An intermediate agent, the platform, matches demand with supply. Agents are individual decision-makers. Specifically, travellers may decide which mode they use or reject an incoming offer; drivers may opt-out from the system or reject incoming requests. All of the above behaviours are modelled through user-defined modules, allowing to represent agents’ taste variations (heterogeneity), their previous experiences (learning) and available information (system control). MaaSSim is a flexible open-source python library capable of realistically reproducing complex interactions between agents of a two-sided mobility platform. MaaSSim is available from a public repository, along with a set of tutorials and reproducible use-case scenarios, as demonstrated with a series of illustrative examples and a comprehensive case study.

## 1 Introduction

Two-sided mobility platforms (like Uber and Lyft) match supply with demand in urban transportation systems. Users submit travel requests in real-time and are matched with drivers, offering to take them to their desired destination. All parties are independent decision-makers acting according to their individual, heterogeneous preferences and learning from past experiences. Travellers are free to select among competing platforms and travel modes. Drivers choose whether to work for the available platforms, freely decide on their working hours and strategically select served requests to maximise revenues. Finally, platforms make strategical decisions to maximise their own profit while being attractive both for the supply (by offering attractive profit for the drivers) and for the demand (by providing services with high reliability and attractive prices).

The emergence of such two-sided mobility markets disrupts the transport landscape. Conventional models for transport planning and operations are focused on top-down planning of service lines, timetables and traffic control measures which are not directly suitable for capturing the double-sided decision-making process and the dynamics of mobility on demand services. A wide array of research questions has consequently emerged, ranging from traffic flow, labour economics, real-time control and optimisation to travel behaviour.

This bursting stream of research calls for the development of a unifying simulation framework under which emerging models, algorithms and approaches may be integrated. Recent changes are disruptive enough to justify a new framework that explicitly accounts for both supply-side and demand-side dynamics, as well as their interaction with the intermediate matching platform.

In particular, in order to capture the bottom-up emerging order resulting from two-sided mobility, it is essential to revise the modelling approach of key elements of the transport system: demand (which is now inherently microscopic), supply (which has become a decision-maker) and a road network (which capacity and congestion are no longer a single pivot variable of assignment models) along with a new agent, the platform, which orchestrates supply and demand interaction and which might be subject to regulation.

Furthermore, the already interdisciplinary field of transportation science has recently gained increasing interest from various fields such as complex network theory, system dynamics, social networks, marketing economics and computational physics. This makes it particularly timely to support a fast learning curve by offering a quick, minimal setup to reproduce the basic dynamics of two-sided mobility platforms. To allow researchers to contribute to their domain, we introduce modular software that requires minimal knowledge of other modules while allowing to enrich the overall experimental analysis.

### 1.1 State of the art and contribution

Understanding and modelling two-sided mobility systems are challenging and require a broad set of interdisciplinary expertise [1–3]. While modelling the relations between the different actors involved in two-sided platforms proved to be non-trivial [4, 5], their manifestation in the context of a dense and congested urban mobility network induces additional complexity.

Since the emergence of the platform economy in the mobility context, empirical evidence has been collected and analysed, revealing new phenomena that call for further, model-based analyses. Prime examples of which include the comparison of services offered by public transport and ride-hailing platforms [6], driver acceptance behaviour [7], drivers’ labour choices [8], Mobility as a Service (MaaS) adoption [9], mode choice [10] and spatio-temporal demand patterns [11].

This, in turn, opens a class of novel research problems arising from the emergence of two-sided mobility platforms including:

- dynamic interactions between supply and demand [12, 13];
- matching of drivers to requests with various optimisation criteria and algorithmic structures [14–17];
- travellers’ mode and platform choices and attitudes towards emerging modes of transportation [18–20];
- drivers’ strategical decision of participation, working shifts and platform choices [7, 21, 22];
- labour economics and the impact of decentralised platform operations [23–25];
- platform pricing strategies, including discriminatory data-driven pricing strategies [26];
- pooled rides, promising in terms of sustainability and efficiency, yet posing significant computational and organisational challenges [27–30];
- fleet operations and management of shared autonomous vehicles [31–34]; and
- re-positioning strategies of drivers and fleet operators [35, 36].

Each of these research domains gives rise to a series of significant and challenging research questions. Answering each of which is non-trivial, yet the main challenge lies in representing the complete system with its (inter)dependencies and feedback loops, non-determinism and adaptive evolution.

In this rapidly developing research field, multiple studies addressed emerging problems by means of simulation frameworks, typically agent-based. Starting from classical taxi operations [37] and extending to emerging modes of car-pooling [38], ride-sharing [39] and ride-hailing [29]. In the absence of an encompassing modelling framework, studies have often been limited to a single aspect. For example, [27] focuses on travellers’ behaviour and neglects fleet operations, whereas [28] focuses on real-time fleet operations neglecting the travellers’ decision process; and [21] relies on an abstract grid network to focus on income equity. Similarly, in STARS [40] travellers are matched with drivers to travel together towards a destination in space and time yet both demand and supply are fixed inputs; in [41] travellers may leave the system if they are unsatisfied, but this is a fixed condition rather than a full behavioural model; while in [42] the day-to-day evolution of both sides is simulated for a fixed within-day behaviour.

More complete solutions typically extend established simulation frameworks. This is most notably the case for SimMobility [43] and MATSim [44]. In [43], supply and demand agents are heterogeneous decision-makers, making daily decisions to participate in the system or not, yet within-day decisions (acceptance) and competition of multiple platforms are not considered. In AMoDeus [44] (which builds on top of MATSim—an urban mobility simulation framework) the focus is on autonomous mobility—while travellers are individual decision-makers, the supply is composed of a fully-controllable self-driving fleet. Both learning and adaptation can be indirectly implemented into AMoDeus via MATSim which allows more detailed demand models (e.g. activity plans) and traffic flow models (to reproduce congestion) but also requires significant effort to implement inside a complex Java environment.

Consequently, we introduce MaaSSim, which is the first modular, extensible framework that contains the fundamental representation of key unique and novel phenomena related to two-sided mobility platforms. MaaSSim is the first publicly available python simulation framework allowing to reproduce within-day and day-to-day dynamics of travellers, drivers and platforms. Key features of a two-sided mobility platform (non-deterministic, adaptive, heterogeneous behaviour of agents interacting with each other) are explicitly handled via user-defined and flexible python functions, allowing to reproduce the desired behaviour and to trace emerging complex dynamics. An extensive set of tutorials and sample experiments facilitates a fast learning curve for users of various backgrounds, while modular, extensible architecture allows for its seamless development.

Notably, MaaSSim is not intended for the complete modelling of transport systems for which there is an abundance of mature and developed frameworks, both commercial (like PTV Visum, CUBE, Emme) and open source (like MatSim [45], SUMO [46], DynaMIT [47], SimMobility [48] etc.). Instead, the explicit objective of MaaSSim is to support researchers with modelling and reproducing the emerging novel phenomena taking place in the context of two-sided mobility platforms and analyse their disruptive potential for urban transport systems.

## 2 Software description

MaaSSim is an agent-based simulator reproducing the dynamics of two-sided mobility platforms in the context of urban transport networks. It models the behaviour and interactions of two kinds of agents: (i) travellers, requesting to travel from their origin to destination at a given time, and (ii) drivers supplying their travel needs by offering them rides. The interactions between the two agent types are mediated by the platform, matching demand and supply. Both supply and demand are microscopic. For supply, this pertains to the explicit representation of single vehicles and their movements in time and space (using a detailed road network graph), while for demand this pertains to the exact trip request time, origin and destination defined at the graph node level. Agents are decision-makers. Specifically, travellers may reject the incoming offer or decide to use another mode than those offered by the mobility platform altogether (opt-out). Similarly, the driver may opt-out of the system (stop providing service) or reject/accept incoming requests. Moreover, drivers may strategically re-position while being idle.

All of the above behaviours are modelled through **decision modules**, the core functionality of MaaSSim. By default agents’ decisions are deterministic and ubiquitous but designed to be easily replaced with user-defined functions representing desired behaviour—presumably probabilistic, representing agents’ taste variations (heterogeneity), their previous experiences (learning) and available information (system control).

**Fig 1:**
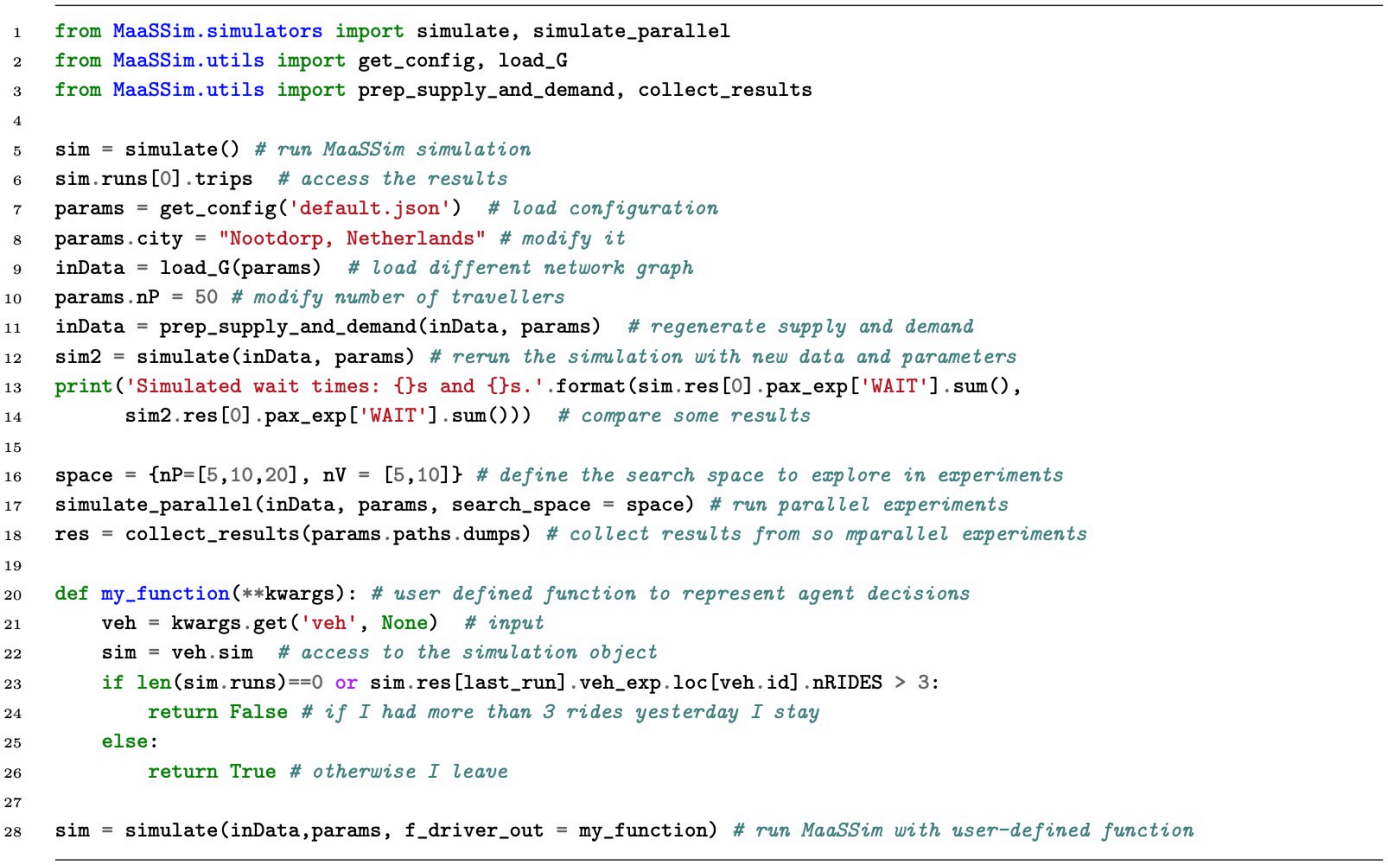
MaaSSim usage at glance: Starting from a single simulation, through modifying input and configuration, up to parallel experiment computation and user-defined decision function.

MaaSSim allows to replicate simulations (to obtain meaningful distributions of random variables), explore multidimensional parameter grids in parallel (e.g. various travellers’ value-of-time and fleet size combinations) or simulate day-to-day evolution until convergence (as we illustrate in the case study of the next section). Independent simulation runs may be executed in parallel, distributing computation load over multiple threads. Each simulation run (day) outputs a sequence of recorded space-time locations and statuses for simulated vehicles and travellers. These outputs are further synthesised into agent-level and system-wide KPIs for in-depth analyses. Fig 1 provides an overview of MaaSSim usage.

### 2.1 Software architecture

**Fig 2:**
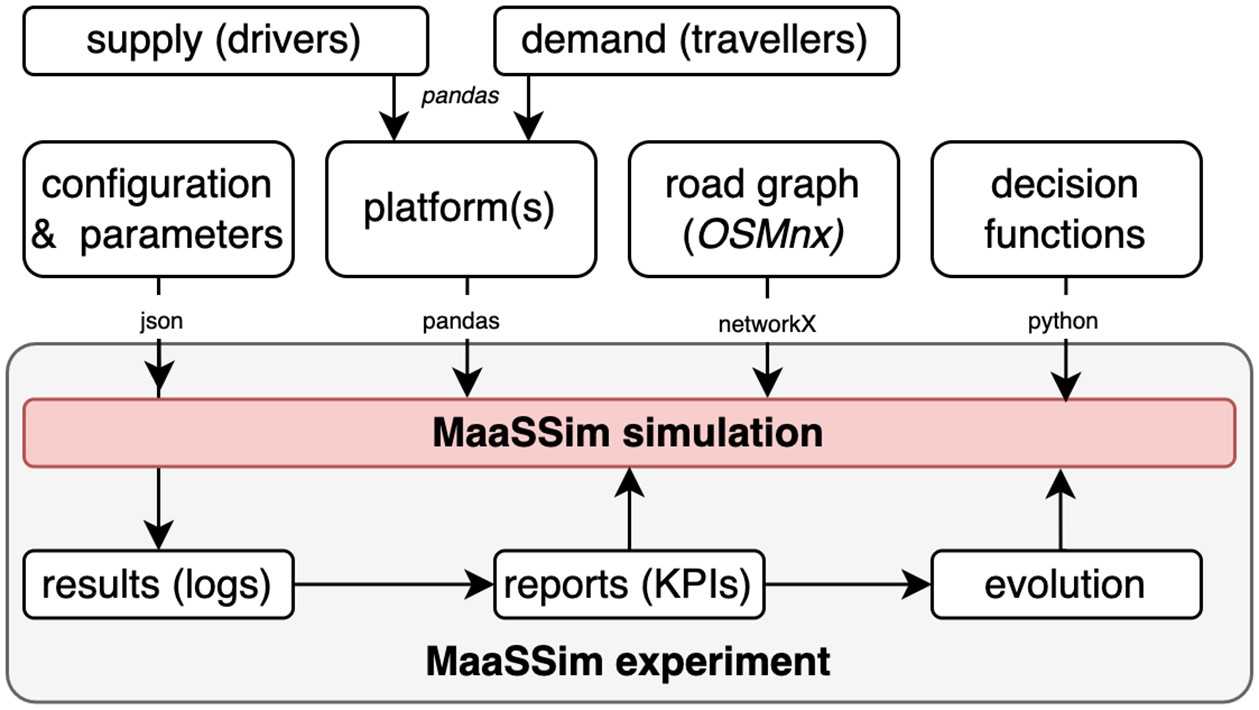
Input and output of MaaSSim workflow.

**Fig 3:**
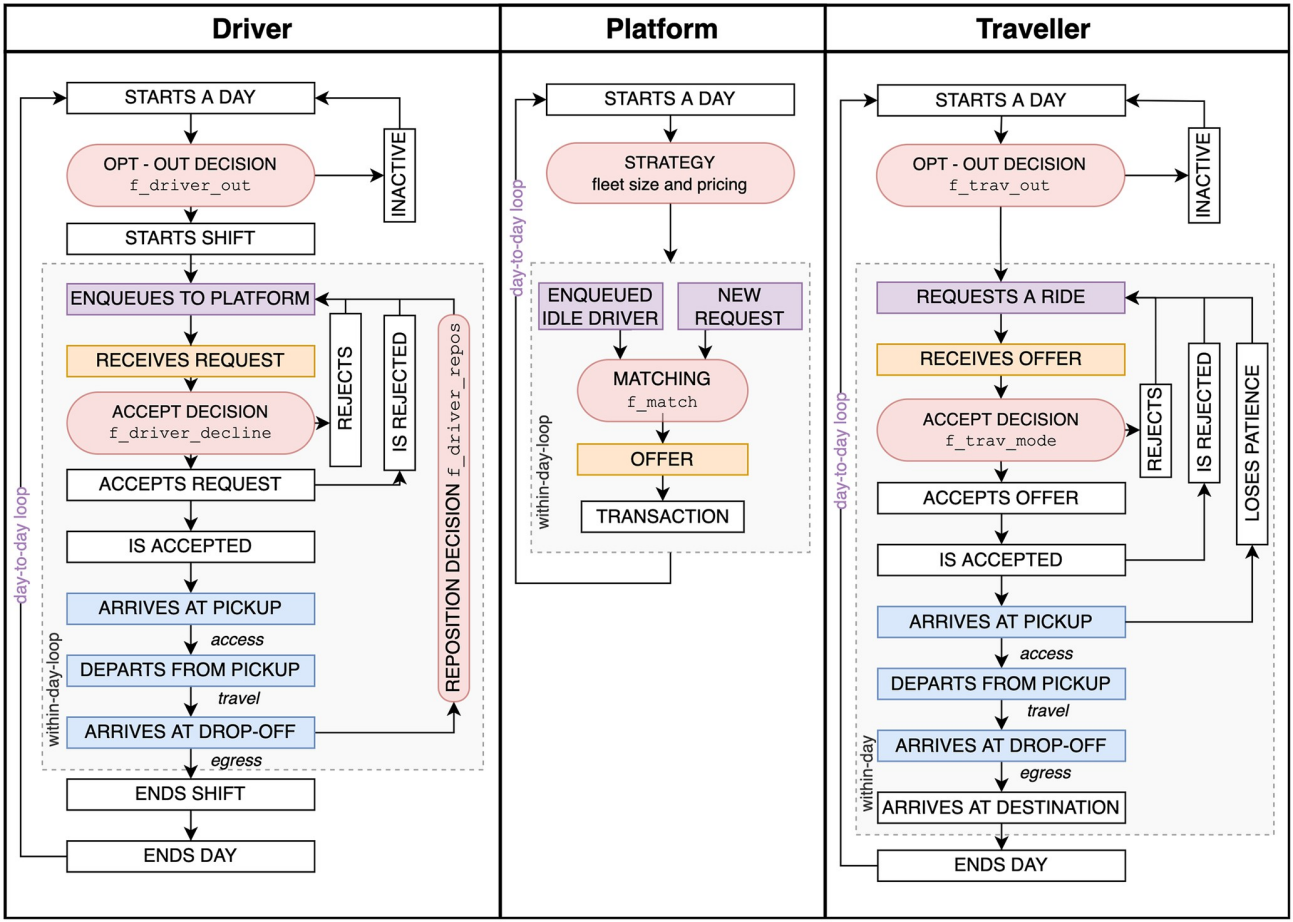
Routines of the three kinds of agents in MaaSSim. Boxes in violet denote an interaction with the platform, orange displays matching between drivers and travellers, and blue refers to the joint part where a traveller is transported by the driver. Places where agents make a decision are marked with red rounded boxes and their decision protocols can be replaced by user-defined python functions.

MaaSSim is a lightweight, modular, scalable and extensible python library. The main class is called with a configuration file (.json file) allowing to control the input (travel demand, fleet supply, road network) and simulation (e.g. simulation time or event duration and their variability). External decision functions to reproduce desired agents’ behaviour are passed by reference and can be user-defined (Fig 2). A simulation corresponds to a single day, during which routines of interacting agents are processed (Fig 3) with a Simpy discrete-event simulation framework [49]. The simulation outputs raw .csv logs where spatio-temporal stamps of consecutive events are stored for each agent, as well as aggregated reports with key performance indicators.

#### 2.1.1 Input

MaaSSim is controlled via an editable .*json* file which configures the simulation and specifies the input. Users may define e.g. supply and demand levels, simulation time, travel speeds and duration of respective simulation events (e.g. transaction time and its variability). A full list of parameters with default values is presented in the Table 1.

**Table 1:** Default parameterization of MaaSSim and parameters’ description.

parameter	name	type	default	description
general	city	String	‘Nootdorp, Netherlands’	query for Open Street Map to download a new graph
	nP	Int	20	demand level (number of trip requests to be generated)
	nV	Int	5	supply level (number of vehicles to be generated)
	nD	Int	1	number of days to be simulated
	t0	String	‘17:00’	simulation start (string interpretable as pd.TimeStamp)
	simTime	Int	1	hours of simulation
times			-	duration of respective events
	request	Int	15	making a request via app
	transaction	Int	20	accepting the request and match
	pickup	Int	30	entering the vehicles
	dropoff	Int	10	leaving the vehicles
	patience	Int	600	maximal time to wait for match before leaving the system
demand density		Float		spatial distribution
	origins		-0.0003	of origins
	destinations		-0.001	of destinations
speeds		Float		mean speeds [m/s]
	walk		1.2	of pedestrians
	ride		10	of vehicles
paths		urls or paths		input files
	G	.graphml	‘Nootdorp.graphml’	osmnx graph
	skim	.csv	‘Nootdorp.csv’	node x node distance matrix
parallel				control parallel simulations
	nThread	Int	1	number of threads
	nReplications	Int	1	number of replications

To run the MaaSSim simulation following input is required:

- an urban road network graph (an instance of networkX DiGraph imported for the simulated urban area with OSMnx [50]),
- travel demand (pandas DataFrame with the origin and destination nodes and departure time for each trip request) and
- supply specifications (drivers with their initial locations).

Both supply and demand may come from external data sources or be generated using internal MaaSSim procedures (e.g. to create synthetic demand patterns following predefined spatial distributions). Each input DataFrame may store additional information in separate columns, interpreted within user-defined modules (e.g. traveller-specific value-of-time or drivers’ working shifts).

#### 2.1.2 Agents

Three kinds of MaaSSim agents (implemented as SimPy processes) interact with each other during the course of their daily routines (Fig 3):

- **Travellers** may be assigned to multiple platforms and submit a request to them to choose the best offer amongst those. A traveller unsatisfied with previous experience may opt-out before requesting. When receiving an offer the traveller makes a decision whether to accept it or not. While accepting she/he walks to the pick-up point, waits until the driver arrives, travels to the drop-off point and walks to the final destination, which terminates the traveller’s daily routine.
- **Drivers** operate in a loop, enqueuing to the platform and serving matched requests until the end of their shift (Fig 3). Drivers may decide to opt-out before starting a shift and not enter the platform at all. When they start their shift, they accept or reject the incoming requests. When accepting, they serve the matched request: arrive at the pick-up point, wait for the traveller, travel to their drop-off point and may reposition after becoming idle.
- **Platforms** operate in an infinite loop during simulation. Every day platform may update its control variables e.g. fare for single and shared rides or a commission fee collected from the drivers. Whenever a trip is requested or a driver becomes idle (starts a shift or completes a previous request), the platform matches a two-sided queue of travellers on one side and drivers on another. By default, incoming travel requests are matched with the nearest idle vehicle. Such a generic algorithm can be easily replaced with a user-defined function (e.g. batching requests—as illustrated with reproducible experiments on the public repository).

#### 2.1.3 Decision modules

The central functionality of MaaSSim lies in representing agents’ individual decision processes (marked with round boxes in Fig 3). To this end, we introduced an interface where default functions may be overwritten with user-defined modules and integrated within MaaSSim simulations. User-defined functions receive as input: the main simulation MaaSSim object and a decision-maker (traveller or a driver along with their individual attributes) which can be used to reproduce the desired behaviour of:

- drivers:
  - leaving the system (f_driver_out),
  - accepting requests (f_driver_decline) and
  - re-positioning (f_driver_repos),
- travellers
  - leaving the system (f_trav_out) and
  - selecting among platforms and modes (f_trav_mode),
- platform
  - updating control variables (fares and commission)
  - matching requests to drivers (f_match)

#### 2.1.4 Computation times

MaaSSim has been developed to facilitate the research and assessment of system operations rather than real-time applications. The focus of its development has therefore been on code clarity and its accessibility for a broad community. Notwithstanding, it remains efficient and allows running real-size computations within a reasonable time. For instance, simulating 1000 travellers and 50 drivers over 4 hours for the city of Delft, the Netherlands takes ca 70s. It requires 28 minutes to simulate the Amsterdam network for 8 hours with 5 000 travellers and 200 drivers, whereas simulating 50 travellers and 5 drivers for the small network of Nootdorp takes less than 2 seconds on MacBookPro 2019. Parallel computations on multiple threads allowed to run 20 000 simulations of an experiment from Fig 5 in less than two hours.

The complexity grows with the number of travellers, drivers and platforms (while each agent adds a new routine, the number of possible interactions between travellers and drivers within the platform follows a quadratic pattern). Surprisingly, network size does not affect the computations, as long as the pre-computed distance matrix fits into memory. The computation times may of course increase significantly if complex decision modules are introduced (and executed along with each agent’s routine).

## 3 Results

We present MaaSSim modelling capabilities through a series of illustrative experiments where we simulate various system settings under a range of configurations, followed by an extensive experimental scheme of more than 1000 simulations of 200 days of supply and demand evolution.

### 3.1 Illustrative examples

The following examples are stored on public repository (https://github.com/RafalKucharskiPK/MaaSSim/tree/master/docs/Experiments to be reproduced and adapted. Below we use the detailed OSMnx network of Delft, the Netherlands (city of ca. 100 000 inhabitants) downloaded from OpenStreetMap with [50]. We set the speed to 36 kilometres per hour across the network. Likewise, the duration of events (transaction time, pick-up time, etc.) and travel speeds are deterministic and fixed. Each simulation day starts with drivers randomly positioned at network nodes. Trip requests connect origins with destinations, both assigned to the randomly selected network nodes. Spatial distributions are set to reproduce a pattern typical to the morning commute, i.e. origins are dispersed, while destinations are concentrated around the centre. Demand is uniformly distributed throughout the analysis period. The ride-hailing platform matches incoming requests with the nearest vehicles. Unless otherwise stated all decisions are deterministic and ubiquitous: travellers and drivers do not opt-out and do not reject incoming matches. Notwithstanding, we assume that travellers leave the system if they have not been matched with a driver after waiting for 10 minutes.

#### 3.1.1 Spatial patterns

**Fig 4:**
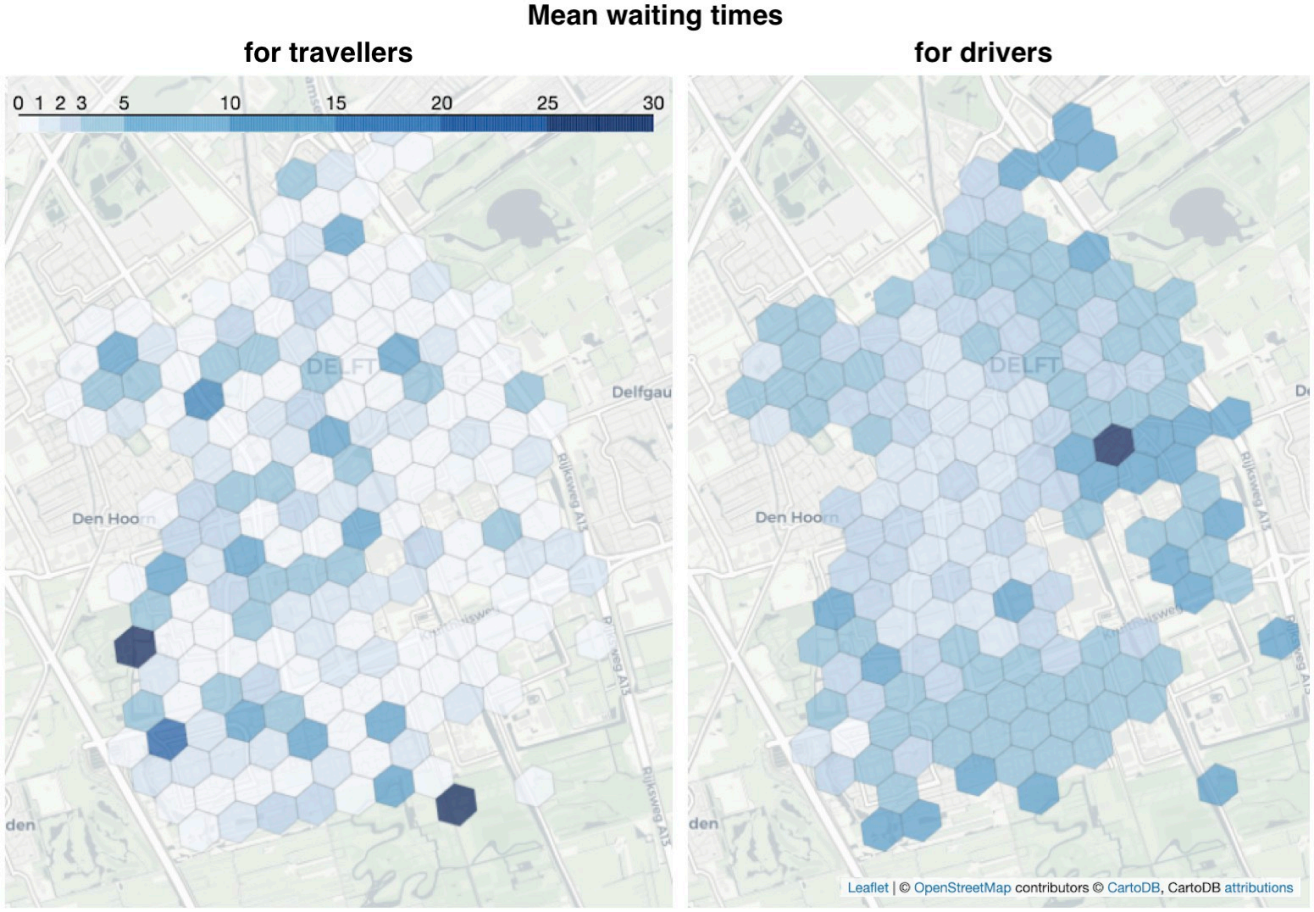
Average waiting time for travellers until the driver arrives (left) and for the driver, until they get requested (right). Dark denotes longer waiting times. Results from 20 replications of a four hour period simulation with 200 travellers and 10 vehicles in Delft, the Netherlands ©OpenStreetMap contributors.

We start with a single simulation, where 10 drivers serve 200 trip requests. A single platform matches travellers to their nearest idle drivers. They meet at the pick-up point and travel together to the destination. We replicate non-deterministic trip requests generation to obtain meaningful results. We report MaaSSim results in terms of waiting times—a key performance indicator for both supply and demand—in the respective parts of the city (hexagons). The obtained spatial patterns reveal conflicting trends (Fig 4). While traveller waiting times (left) are low in the Eastern parts and high in the Western parts of Delft, for drivers the opposite trend prevails (right). This reveals an interesting interaction between agents and potentially conflicting interests in the two-sided mobility market, similar to the ones observed empirically e.g. in Philadelphia [51] or in Beijing [11].

#### 3.1.2 Supply and demand interactions

**Fig 5:**
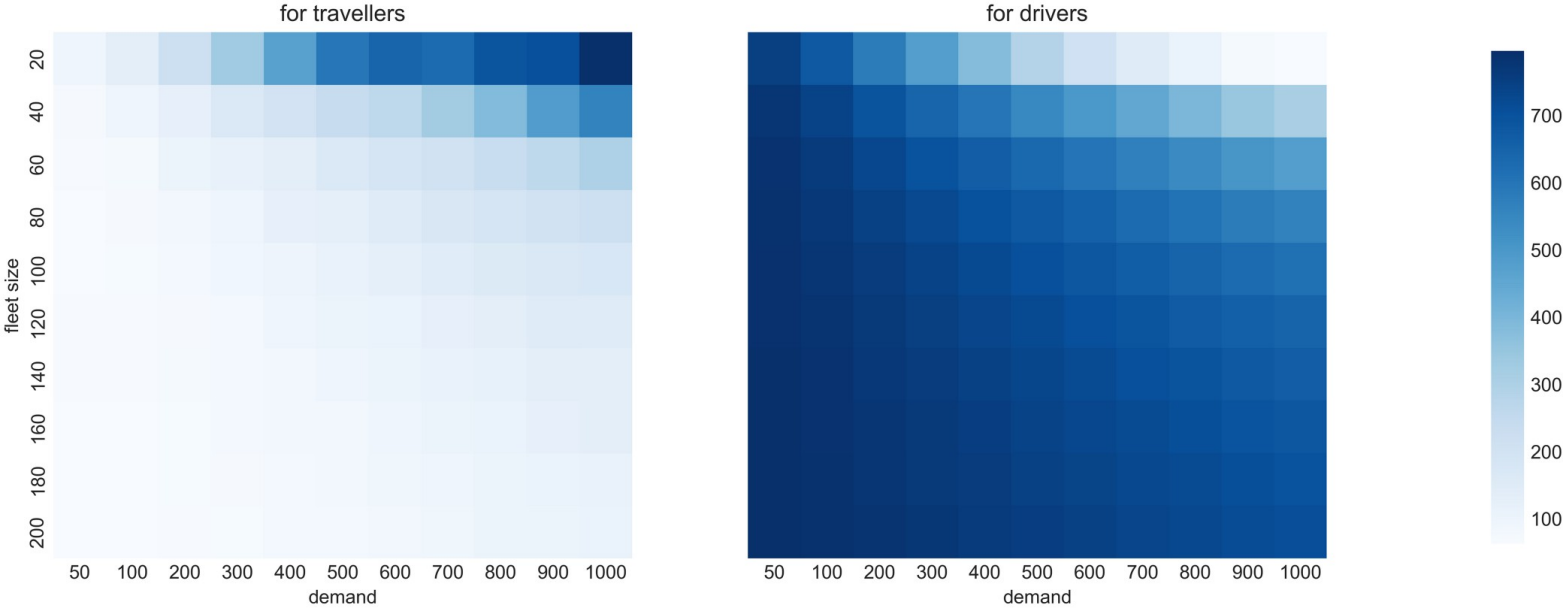
Service performance for various demand and supply levels. Average waiting times for travellers (left) and drivers (right), which follow mirroring diagonal trends.

Next, we examine the supply and demand interactions in various settings. We explore the scenario grid varying from low to high demand (trip requests per hour) and supply levels (vehicles serving requests). The waiting time for travellers (Fig 5 left) is low when there are few travellers and many drivers. Conversely, drivers’ idle times decrease if the fleet size is low (Fig 5 right). While this overall trend is expected (and supports empirical findings e.g. from [52] and [53]), the magnitude and the sensitivity of these relations and their potential to result in feedback loops on both the demand and supply sides of the two-sided market would not have been possible without detailed modelling with MaaSSim.

#### 3.1.3 Platform competition

**Fig 6:**
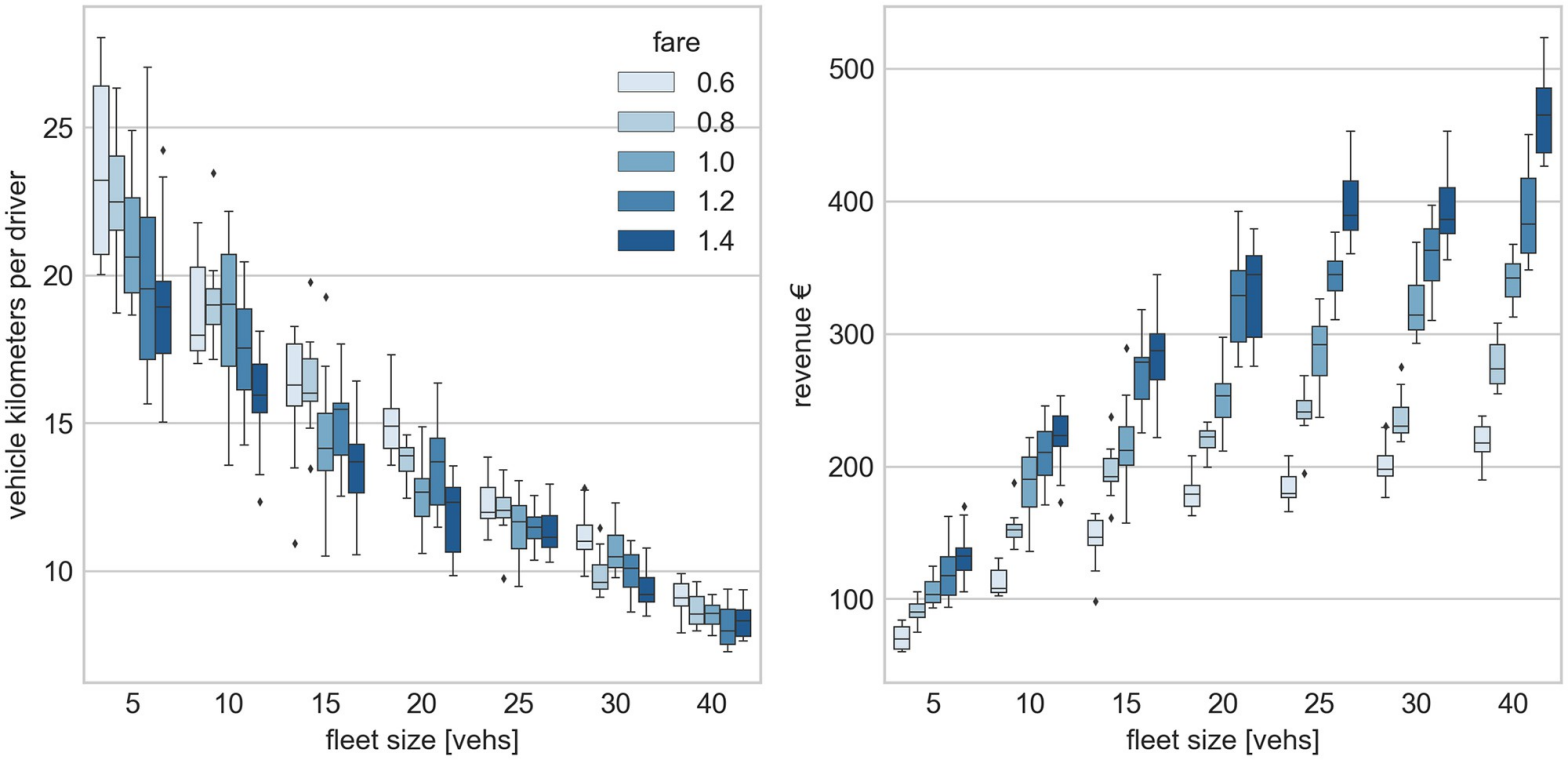
Searching for optimal platform competition strategy: A platform enters a market with a competitor operating a fleet of 20 vehicles and offering a trip fare of 1.0 unit/km. We report average vehicle kilometres per driver (left) and total platform revenues (right) resulting from varying fleet size (x-axis) and fare (per-kilometre) combinations for 20 replications.

Such interaction between the supply and demand typically leads to questions about the strategic behaviour, reinforced learning and system equilibria, like in the empirically observed platform competition between Uber and Lyft in the United States [54]. We illustrate how MaaSSim supports answering those questions by means of a platform competition experiment. We consider a system where an existing platform with 20 drivers offers its travellers a trip fare of 1.0 units/km. We explore potential strategies for a new platform entering the market in terms of two key variables: fleet size (varying from 5 to 40 drivers) and a fare (varying from 0.6 to 1.4 units/km). Fig 6 shows the mileage per driver (left) and the total platform revenues (right) resulting from the combination of various strategies. The box plots denote means and distributions resulting from 20 replications.

Since in the above experiments the simulations are independent (unlike the reinforced learning, where agent decisions are dependent on their experience learned from previous simulation runs) we use parallel computations on multiple threads and collect results in a single file for analysis (which can be replicated with publicly available code).

#### 3.1.4 Drivers learning

**Fig 7:**
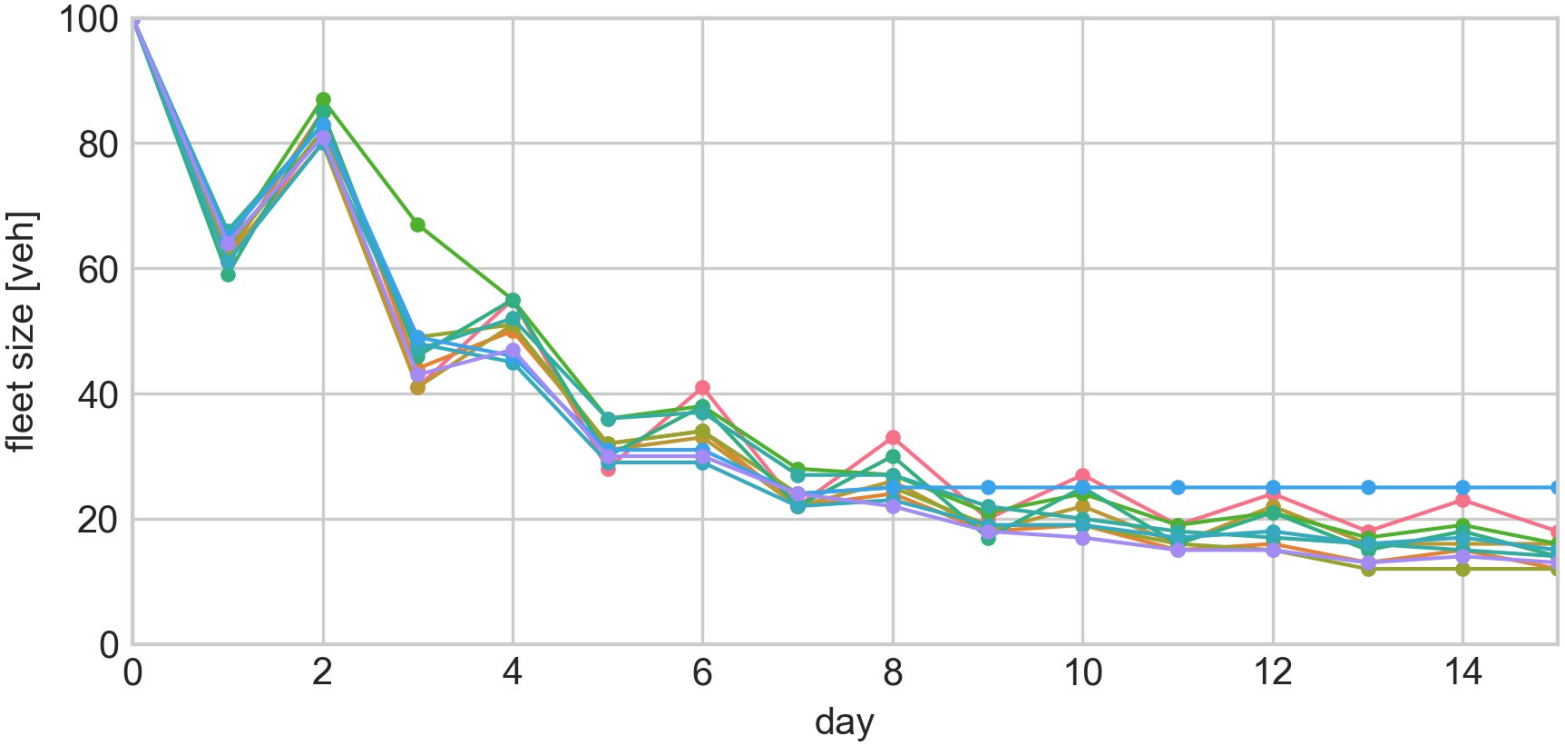
Fleet size evolution for 10 non-deterministic replications of driver learning behaviour. Drivers make daily decisions to opt-out or stay in the system based on previous experience and expected outcomes.

We illustrate the strategic learning of agents with a scenario where 100 drivers serve 200 travellers in a sequence of day-to-day simulations (Fig 7). Apparently, the initial supply level is too high, resulting in short waiting times for travellers and low revenues for drivers. Unsatisfied drivers will opt out due to bad previous experiences (low income), adjusting the pool of drivers which decreases until, eventually, some drivers decide to return to the system (as they observe high revenues in the adjusted system). This decision process is modelled via a user-defined python function (see example in Fig 1) which can introduce any generic formulas (e.g. discrete-choice model), parameters (also from .json configuration file) and agent-specific attributes (e.g. individual value of time). Such behavioural adaptation leads to system stabilisation, which may vary due to non-deterministic simulation settings (demand distributions and initial vehicle positions). A similar process was reported e.g. by [55] where drivers successfully adapted to maximise their revenues.

#### 3.1.5 Ride-pooling

**Fig 8:**
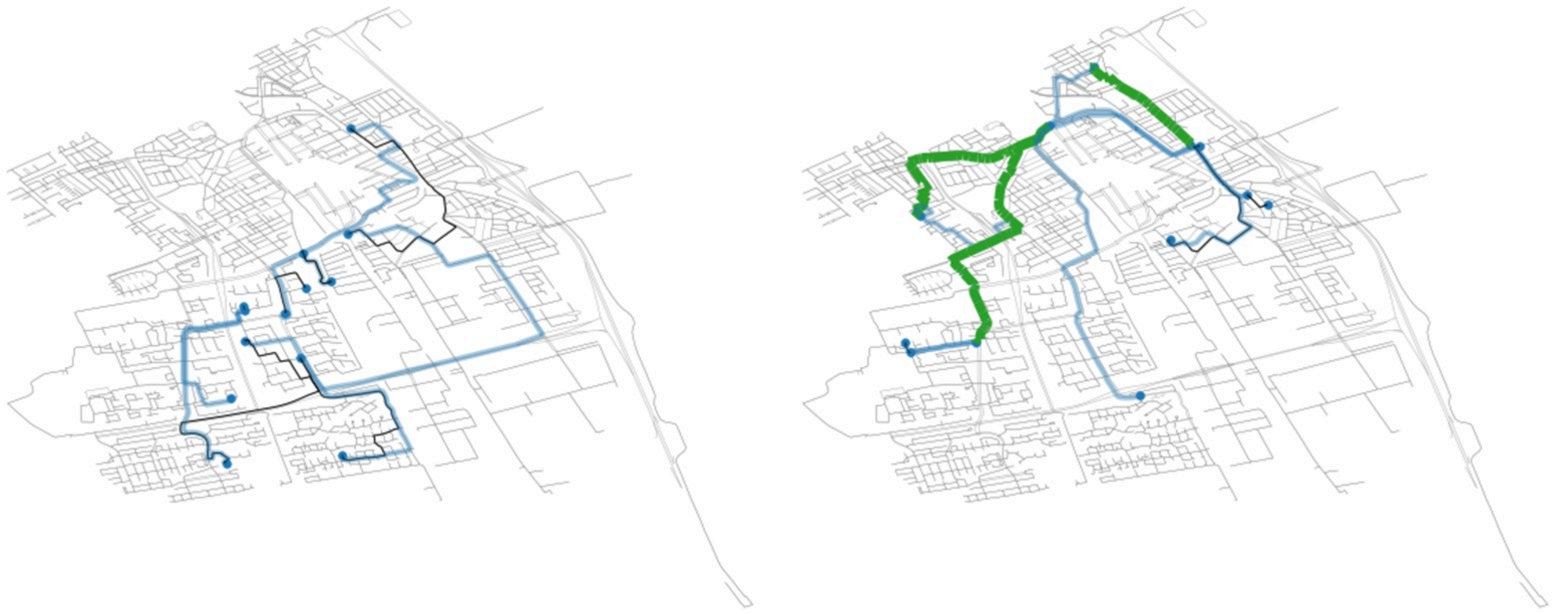
Traces of rides for a single simulated vehicle without (left) and with pooled ride services (right). Blue—single traveller on-board, Green—several travellers sharing a ride, Black—empty vehicle trip.

Finally, we demonstrate how ride-pooling is embedded with simulations in Fig 8, where we show a trajectory of a vehicle serving non-shared, private rides (left) and pooled-rides (right). Shared rides are here pre-computed with external the ExMAS [27] algorithm. ExMAS is fully integrated in MaaSSim so that ride-pooling can be easily reproduced, as demonstrated in the online tutorial: https://github.com/RafalKucharskiPK/MaaSSim/blob/master/docs/tutorials/07_Shared_rides_with_ExMAS.ipynb. relying on behavioural and system parameters to optimally match travellers into attractive pooled rides. In this study, we use a 20% discount for a shared ride and 1.2 penalty for the shared in-vehicle time (so-called willingness to share multiplier), further detailed in [27]). With such a setting, one can reproduce the impact of ride-pooling on system efficiency, revealed e.g. in Chicago [56].

### 3.2 Case study

**Fig 9:**
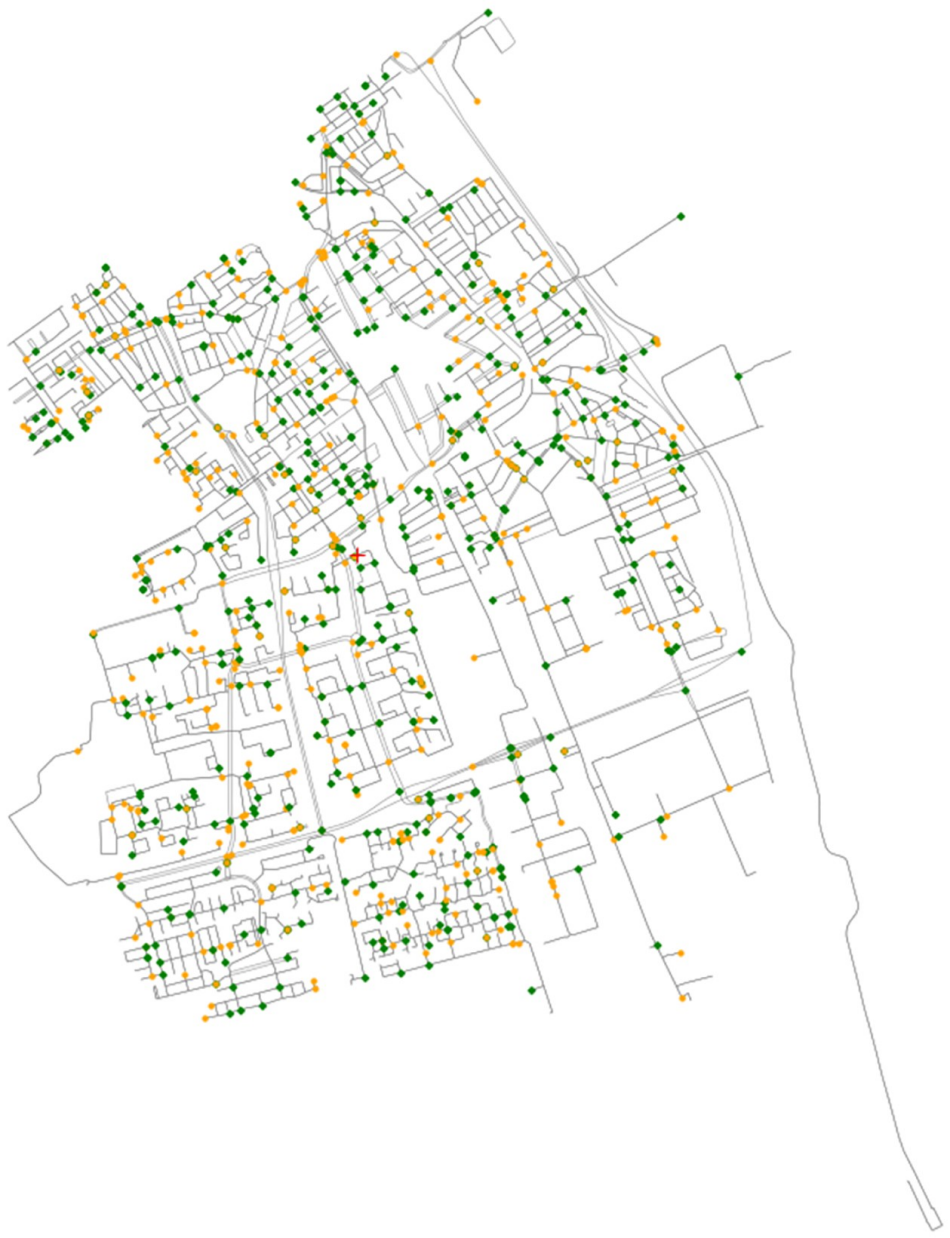
Road network of Delft with origins marked in green, destinations in orange. ©OpenStreetMap contributors.

To demonstrate the full complexity of two-sided market dynamics and MaaSSim capabilities to reproduce it, we report here a comprehensive simulation experiment. We simulate demand and supply evolution governed by the platform decisions in the city of Delft, the Netherlands. Drivers start each day at their fixed locations and travellers enter the system with the same travel demand (origin, destination and departure time)—as illustrated with Fig 9. Travellers are matched by the platform to the nearest drivers and together they traverse a detailed road network graph.

Every day travellers make travel choices. Based on their past experience they select between three alternatives: private ride-hailing (*rh*), ride-pooling (*rp*) or other alternative modes (e.g. public transport, car or bike). Which depends on their expected quality of service, i.e. waiting time, travel time and trip fares. Similarly, the drivers every day decide whether to participate in the system or not, relying on their past experiences. They compare their expected wages with the so-called reservation wage and make rational decisions (analogously to [57]).

Eventually, the learning process terminates as the system converges to a stable state. The evolution depends not only on the supply and demand levels but is also controlled by the platform strategy. The platform controls the behaviour of drivers by determining the share of collected fare that stays with a driver *c* and influences the behaviour of travellers by setting the discount rate for ride-pooling *p* (as an incentive to induce pooling)—both are treated in the experiments as exogenous variables, directly controlled by the platform.

The day-to-day decisions are modelled using microscopic probabilistic discrete choice models, in which agents (travellers/drivers) estimate their utility associated with each alternative and make subjectively optimal decisions. These decisions are implemented as user-defined python functions passed to MaaSSim at the initialisation and controlled via a .json configuration file.

Drivers update their expected profit (wage *w* of driver *d* on the day *i*) based on their experiences:

$$\begin{array}{c} {\bar{w}}_{i,d}=f\left({\bar{w}}_{i-1,d},w_{i-1,d}\right) \end{array}$$ (1)

Their expectation ($\bar{w}$) is updated based on the actual experiences (*w*) from previous days and used to make subsequent travel decisions (similarly to [55]). The probability of participating (driving) in the system is then expressed as:

$$\begin{array}{c} p_{i,d}=\frac{e^{\beta_{d}{\bar{w}}_{i,d}}}{e^{\beta_{d}{\bar{w}}_{i,d}}+e^{\beta_{d}W_{i}}}, \end{array}$$ (2)

where the expected wage is compared with a so-called reservation wage *W*_*i*_ in a binary probabilistic choice model with a sensitivity parameter *β*_*d*_ (set to 0.1 in the experiment). The reservation wage *W*_*i*_ is user-dependent and drawn for each driver from a normal distribution with a mean of 10€ per simulation hour and a standard deviation of 2.5€. We store it as the extra column in the input DataFrame and use in further calculations.

Travellers choose between three alternative travel modes, following a multinomial logit model:

$$\begin{array}{c} p_{i,d}^{m}=\frac{e^{U_{i,d}^{m}}}{e^{U_{i,d}^{h}}+e^{U_{i,d}^{s}}+e^{U_{i,d}^{o}}}, \end{array}$$ (3)

where the probability that individual *i* chooses mode *m* on day *d* is calculated based on the utility of this mode, relative to the utilities associated with alternative modes (similarly to [10]). Here, we consider three modes: private ride-hailing (*rh*), shared ride-pooling (*rs*) and other, non-platform based modes (bike, public transport and car). Mode utilities are based on travellers’ preferences (expressed with *β*’s in the utility formulas) and travel attributes (travel time *t*, cost *c* and waiting time *w* of respective mode *m*):

$$\begin{array}{c} U_{i}^{m}=\beta_{w,i}w_{i,m,d}+\beta_{t,i}t_{i,m,d}+\beta_{c,i}c_{i,m,d} \end{array}$$ (4)

The attractiveness of platform-based modes is controlled by the price, set by the platform. The dynamics in the choices are induced by accumulating acquired experienced which is then used to form expectations regarding travel times *t* and waiting times *w* of both private and pooled rides. The waiting times depend mainly on the supply (available vehicles) which, in turn, is controlled by the platform through the commission fee (a low commission fee will attract more drivers). Even though travel times are assumed fixed (fixed network-wide speed) in these series of experiments, the detour induced due to ride-pooling may vary and is unknown to the travellers. We specify the behavioural parameters (*β*’s) based on a recent stated-preference study [10], tuned to induce greater market shares (assuming critical mass for efficient platform-based operations is already reached) and greater sensitivity to waiting time. Under this setting, the share of platform-based modes varies from ca. 50% for null waiting times, to 10% when the average waiting time equals 5 minutes.

Travellers are initialised with optimistic expectations of travel and waiting times. In the course of their learning, they gain experience and update their expectations accordingly.

Agents update their individual expectations based on their recent experience using the following formula:

$$\begin{array}{c} {\bar{t}}_{i,d}=\left(1-\omega_{i,d}\right)\cdot {\bar{t}}_{i-1,d}+\omega_{i,d}\cdot t_{i,d} \end{array}$$ (5)

The weight *ω*_*i*,*d*_ depends on the number of experiences that the traveller has acquired *H*_*i*,*d*_ and is bounded by a so-called look-back window (*ω*_*max*_—the number of days used to update the experience):

$$\begin{array}{c} \omega_{i,d}=1/\text{min}\left(H_{i,d},\omega_{max}\right) \end{array}$$ (6)

**Fig 10:**
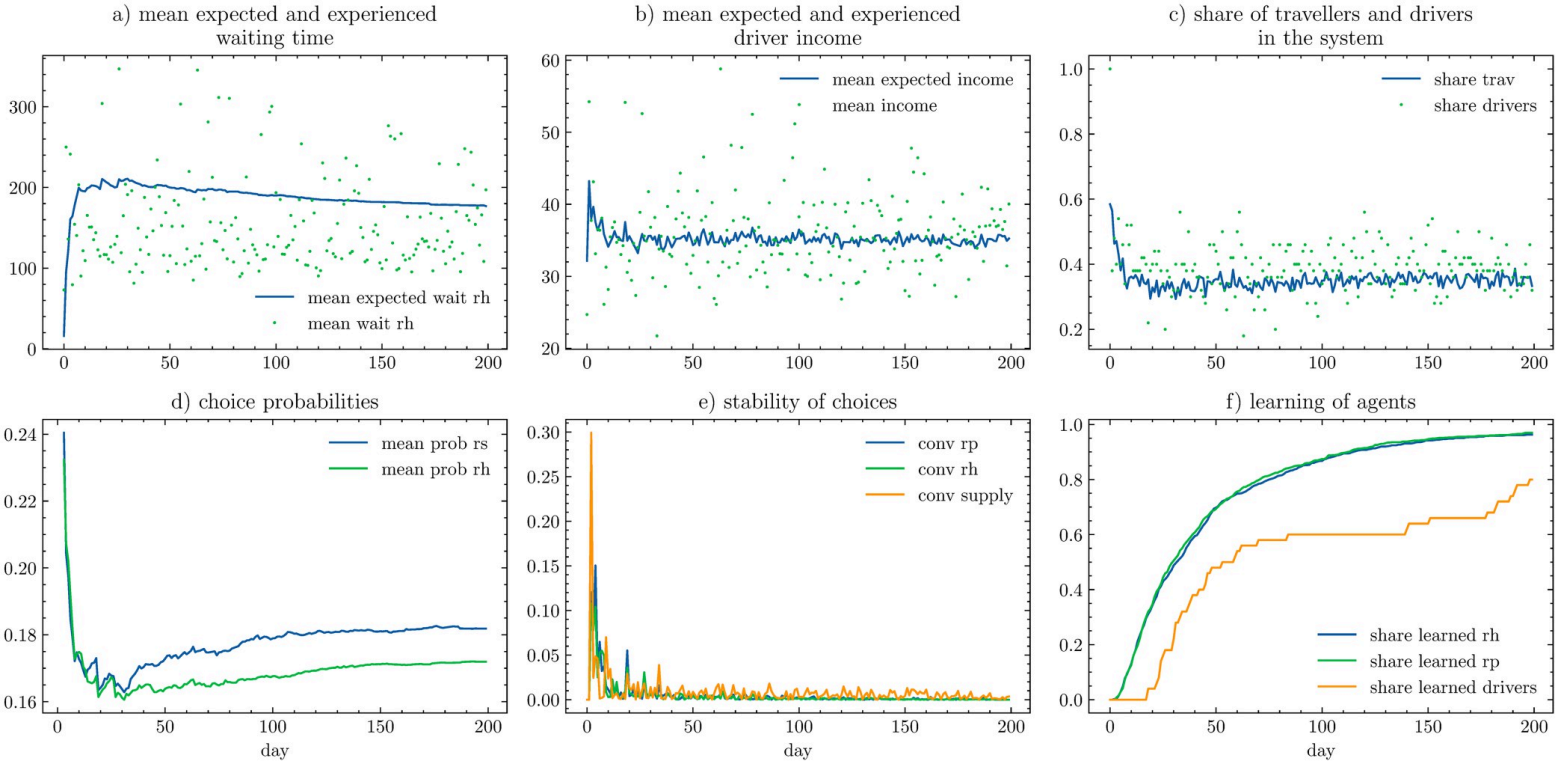
200 days of supply and demand evolution for the case study experiment with 900 travellers and 50 drivers. Travellers expected waiting time (a) stabilises over consecutive days (blue line) despite the high day-to-day variability of actually experienced waiting times (green dots). Similarly for the drivers, whose mean expected incomes (b) remain stable around 35€, with day-to-day variability (green dots) ranging from 27 to 50€. The share of travellers in the system (c) remains stable around 37% (blue line) yet the number of drivers (green dots) may range between 10 and 30%. The initially high choice probabilities for MaaS modes (d) happened to be over-optimistic and quickly drop from 24% to 16%. Yet, as agents learn, the pooled alternative (blue—*rs*) becomes more popular than private ride (green—*rh*) stabilising around 18%. Agent day-to-day choice evolution stabilises and the variability stays within the 2% range already after ca. 40 days of evolution (e). After 100 days 90% of travellers have gained enough experience to learn the system and stabilise their choices (blue and green in the panel f). However, drivers learn slower with only 60% of them finishing the learning process after 100 days (orange on panel f).

**Fig 11:**
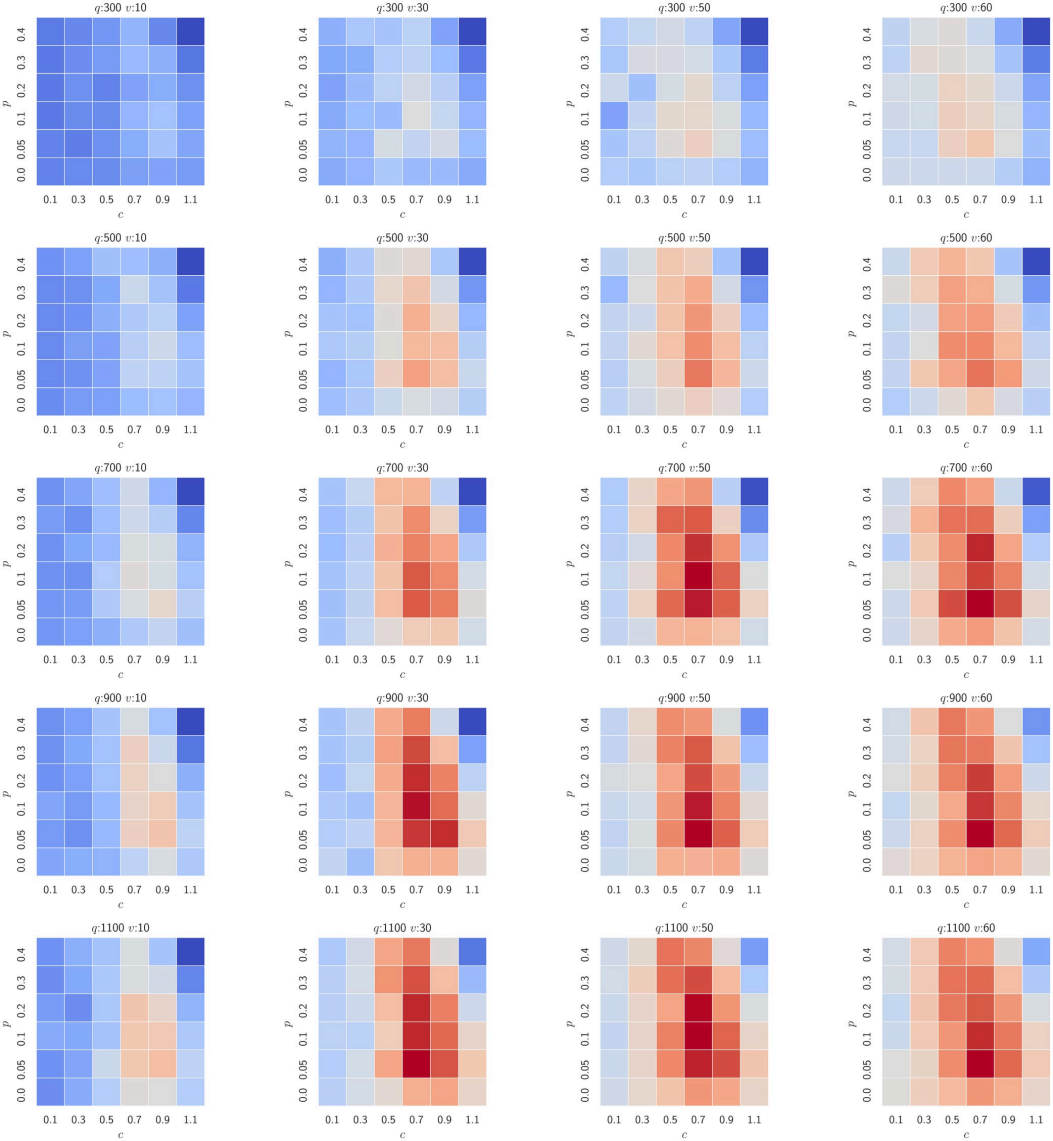
The total commission collected by the platform in the series of over 1000 experiments (blue cells denote deficit, red ones denote profit). Each grid denotes a single supply-demand setting with a fixed number of trip requests (*q*) and a fixed number of potential drivers (*v*). Every single cell therein represents a stable state of the evolution process depicted in Fig 10. The platform explores the best setting of a ride-pooling discount (*p*—rows) and a share of fares remaining with a driver (*c*—columns) to maximise its profit. For a low demand the system is not profitable (top left), yet as the number of drivers increases, the platform starts generating profits (top right). As both supply and demand increase, the central parts of the grids become profitable, i.e. commissions between 0.5 and 0.7. Notably, a too low commission for drivers (<0.5) is not profitable for the platform, as it leads to fewer participating drivers and thus lower revenues, and so does a too high commission for drivers (when drivers stay within the system but the platform does not generate profit). At the high supply and demand levels, the platform may start providing pooled rides as it remains profitable even for high discounts offered (*p* > 0.2). Nonetheless, under this setting the platform profit is always maximised at low discounts (*p* = 0.05) and introducing lower prices for pooling would result in lower profits.

We explored the grid of the following parameters in the experiments:

- demand 300, 500, 700, 900 and 1100 trips per 4 hours of simulation.
- supply 10, 30, 50, 60 vehicles
- share of fares collected by the driver 10, 30, 50, 70, 90, 110%
- pooling discount 0, 5, 10, 20, 30, 4%

and let the system evolve until the stabilisation (when agents finish their learning process). The results are presented and discussed with the sample of the evolution process in Fig 10 and the platform profits on the search space grid in Fig 11.

## 4 Conclusion

The overarching objective underlying the development of MaaSSim is to allow researchers to focus on their partial models and integrate them within the simulation framework, allowing a group of interdisciplinary researchers to share expertise from their fields. For instance, MaaSSim has been instrumental in the scientific discovery process of supply-side dynamics and the fleet size attained in equilibrium in a decentralised bottom-up mobility platform context. This research resulted in a novel service supplier (i.e. driver) learning and choice model [57] which can be now reused for studying platform pricing strategies, re-positioning algorithms or travellers’ mode-choices.

MaaSSim provides an extensible, easy-to-use simulation platform allowing for user-defined representation of a two-sided mobility platform that can support a variety of research interests. MaaSSim is an open-source library available through the pip installer as well as from the public repository, where it comes along with a set of tutorials and applicable use-cases. With the above set of experiments, coupled with reproducible jupyter notebooks stored on the repository, the reader can get an impression of the range of MaaSSim applications and start developing their own experiments. In particular, explore their own networks (queried from OpenStreetMap with [50]) and run the experiments with tailored configurations. Making it capable to support researchers in exploring future research directions in the field of two-sided mobility platforms.

As always, the realism of the obtained simulation outputs strongly depends on the quality of the input parameters. MaaSSim can be parameterized to reproduce a variety of system representations, yet the empirically valid settings remain largely unknown due to the limited availability of data and evidence from the rapidly evolving ecosystem of two-sided mobility platforms. While the active stream of research contributes to a better understanding of the underlying phenomena, additional empirical analysis is needed to better underpin some of the simulator parameters.
